# Comparison of the Efficacy of Dexamethasone and Methylprednisolone in Infiltration Injection for Postendodontic Pain in Patients with Necrotic Pulp: A Randomized Controlled Clinical Trial

**DOI:** 10.1155/2022/4163120

**Published:** 2022-02-23

**Authors:** Sheyda Yeganegi, Nafiseh Fazelian, Mohammad Karim Layegh Nejad, Leila Manzouri

**Affiliations:** ^1^Dental School, Yasuj University of Medical Sciences, Yasuj, Iran; ^2^Department of Restorative Dentistry, Dental School, Bushehr University of Medical Sciences, Bushehr, Iran; ^3^Department of Endodontics Dentistry, Dental School, Yasuj University of Medical Sciences, Yasuj, Iran; ^4^Social Determinants of Health Research Center, Yasuj University of Medical Sciences, Yasuj, Iran

## Abstract

**Purpose:**

Postendodontic pain is one of the problems of root canal therapy. This clinical study aimed to evaluate the effect of infiltration injection of dexamethasone and methylprednisolone on postendodontic pain in patients with necrotic pulp.

**Methods:**

A total of 80 volunteers with necrotic pulp teeth were included and assigned to two groups (*n* = 40). After the administration of local anesthesia and before root canal therapy, in group 1, an infiltration injection of 1 ml of dexamethasone was done and in group 2, an infiltration injection of 1 ml of methylprednisolone was done in the buccal vestibule of each tooth. Patients' pain was reported using a visual analogue scale at pretreatment and 6, 12, 24, and 48 hours after treatment.

**Results:**

There was no significant difference between the two groups receiving dexamethasone and methylprednisolone at pretreatment and 6, 12, 24, and 48 hours after endodontic treatment.

**Conclusions:**

Infiltration injection of dexamethasone and methylprednisolone had a significant effect in reducing pain after the endodontic treatment in necrotic pulp teeth, but between 6 and 12 hours, methylprednisolone had significantly more effect on pain relief than dexamethasone. Overall, the use of any of these drugs to reduce postendodontic pain is recommended.

## 1. Introduction

Despite the advances in root canal treatment, endodontic postoperative pain is common and can occur between 3% and 58% of patients depending on the variables: host-related factors, such as patient's age, gender, tooth type, psychological and immunological factors; the iatrogenic factors, such as over instrumentation and chemical or obturating material; and the microbial factors. Proper anesthesia techniques reduce pain during treatment, but postendodontic pain is still a problem [1].

Clinical signs and symptoms and the radiological findings of the treated tooth determine the success or failure of endodontic treatment. The success symptoms are the absence of pain, inflammation, and fistulas and the maintenance of the functional tooth [2]. Although following endodontic treatment, most patients only experience mild discomfort, up to 6% of patients have experienced more severe pain. Postoperative pain is most intense in the first 48 h following treatment and progressively decreases over the next few days [3].

In necrotic teeth, the passage of infected debris into the periapical area causes postoperative endodontic pain [4].

Several strategies are suggested to manage or prevent postoperative endodontic pain. Several pharmacological (use of analgesics, sedative-hypnotics, mood modifiers, and anesthetics) and nonpharmacological methods (acupuncture, hypnosis, and electrical stimulation) have been used to treat and relieve postendodontic pain. One of the suggested methods to control pain is to prescribe medications before endodontic treatment (premedication) [5].

Postoperative endodontic pain is contributed to inflammatory mediators such as prostaglandins that activate sensitive nociceptors and have a key function in the pathogenesis of periradicular and pulpal diseases [6].

Nonsteroidal anti-inflammatory agents and steroids have been used to interfere with inflammation and to prevent or stop endodontic pain [7].

In some severe and uncontrollable postoperative endodontic pain, it seems that the prescription of steroidal anti-inflammatory drugs is a suitable treatment [8].

Corticosteroids are the most effective anti-inflammatory therapy and they suppress inflammation [9].

Dexamethasone is a long-acting glucocorticoid that has more anti-inflammatory activity than other steroids. For example, the relative potency of dexamethasone is 25 times larger than that of hydrocortisone and 5 times larger than that of methylprednisolone [10] which has a biological half-life of 36 to 54 hours [11]. Methylprednisolone is an intermediate-acting glucocorticoid that is distributed immediately after oral administration. Its biological half-life is 18 to 36 hours [11].

Some studies were done to evaluate the effect of corticosteroid on postendodontic pain in teeth with irreversible pulpitis. Studies by Yavari et al. [12], Aksoy and Ege [13], Yousaf et al. [14], and Naseri et al. [15] on the effect of injection of corticosteroids on postendodontic pain in teeth with symptomatic irreversible pulpitis showed the effect of corticosteroids on decreasing pain after endodontic treatment.

The study by Moradi et al. on the efficacy of infiltration injection of dexamethasone on postendodontic pain of necrotic teeth showed infiltration administration of dexamethasone did not reduce postoperative pain severity in the first 12 hours after endodontic treatment [16]. Because few studies have evaluated the effect of local infiltration of corticosteroids on postendodontic pain in necrotic teeth, the present study aimed to evaluate the efficacy of infiltration injection of dexamethasone and methylprednisolone on postendodontic pain in patients with necrotic pulp. The null hypothesis was that there is no difference in postendodontic pain in patients with necrotic pulp between dexamethasone and methylprednisolone.

## 2. Materials and Methods

### 2.1. The General Data

After taking approval from the ethical committee (IR.YUMS.REC.1396.136), this randomized clinical study was conducted in the endodontic department of dental school. Inclusion criteria were as follows (*n* = 80): (1) patients with good general health (ASA 1 or 2 health status is 14), (2) patients who were willing to participate, (3) patients who have not received anti-inflammatory or analgesic drugs in the last 8 hours, (4) patients who have not taken any drugs, (5) patients who have not an allergy to dexamethasone and methylprednisolone and (6) aged between 18 and 60 years, and (7) with necrotic tooth without periapical lesions [13].

Exclusion criteria were as follows: (1) patients who have systematic complexity (ASA III, IV, and V), (2) patients who were unwilling to cooperate; (3) patients who have taken drugs, and (4) patients whose treatment was more than one session.

After taking informed consent, 80 patients of both genders with good general health were selected for this study using a convenient sampling technique and were randomly divided into two equal groups with the help of a scientific random number table. All participants who fit the criteria volunteered, received verbal and written descriptions of the study design, and signed informed consent forms.

The sample size was based on the study of Jalalzadeh et al. [17]. With a level of significance of 5%, with the power of the test kept at 95%, a total sample size of 80 patients was calculated. Population proportion *A* (no report of pain in patients receiving dexamethasone) = 60%; population proportion *B* = 30% difference in pain relief in treatment with methylprednisolone (dexamethasone is stronger than methylprednisolone).

### 2.2. Clinical Technique

Demographic information, medical and dental history, relevant clinical examination, and periapical radiographs were taken, and all data (including age, sex, pulpal status, pain intensity, and duration of pain) were recorded on the patient chart. The study procedure was explained to each volunteer, and they were divided into two groups, randomly (*n* = 40). In two groups, pulpal anesthesia of mandibular molars was done with the alveolar nerve block technique by injections of 1.8 mL (equivalent to 1 cartridge) of 2% lidocaine with 1/100000 epinephrine (Nova DFL, Rio de Janeiro, Brazil), and for maxillary molars, 1 cartridge of 2% lidocaine with 1/100000 epinephrine was injected by infiltration.

Then in group 1, infiltration injection was performed using 0.2 mL of dexamethasone (8 mg/2 mL, Sigma, St. Louis, MO, USA) with similar conditions and devices of infiltration anesthesia.

In group 2, infiltration injection was performed using 0.2 mL of methylprednisolone (8 mg/2 mL, Sigma, St. Louis, MO, USA) with similar conditions and devices of infiltration anesthesia. The reason for not injecting a placebo in this study was ethical considerations.

As this study was accomplished in a double-blinded manner, the drugs were coded A and B.

After 15 minutes in mandibular teeth and 7 minutes in maxillary teeth, root canal treatment was initiated under rubber dam isolation. Working length within 2 mm shorter from radiographic apex was taken with apex locator. The root canals were instrumented with S1-S2-F1-F2 ProTaper rotary files (Dentsply, USA). The canals were irrigated with 2.5% sodium hypochlorite (NaOCl), dried with paper point and obturated with laterally condensed gutta-percha (DiaDent Group International, Cheongju, Korea) and sealer (AH-26, Dentsply, Tulsa Dental, Tulsa, OK, USA). The access cavity was restored with temporary restoration (Cavit, ESPE-Premier, Norristown, PA, USA), and to prevent future pain, the occlusion and proximal contact were checked. All procedure was completed by one root canal specialist use of single-visit endodontics treatment [18].

The patients were also asked to record the type and dosage of analgesic consumption if required to relieve their postoperative pain. The pain intensity was measured by a nonnumeric VAS ruler. According to this scale, the level of pain was recorded by the operator in the range of 0–170 [19]. A score of 0 indicates no pain, a score of 1 to 54 indicates mild pain, a score of 55 to 114 indicates moderate pain, and a score of 115 to 170 indicates severe pain. Patients were instructed to complete a pain diary on VAS at 6, 12, 24, and 48 h intervals after root canal instrumentation [18].

### 2.3. Statistical Analysis

Statistical analysis was performed using SPSS version 16. Mean, standard deviation, and frequency distribution were measured for each group. Chi-square test, independent-samples *T*-test, and ANOVA test were used to evaluate the study data. The level of significance was set at 0.05.

## 3. Results

In this study, 80 people were studied. The mean and standard deviation of the participants was 30.13 years. The minimum and maximum ages were 18 and 47 years, respectively, and the mean age was 28 years. The mean age in the two groups was not statistically significant. The frequency distribution of gender in the two groups was not statistically significant. The mean and standard deviation of pain duration was 1.42 ± 3.34 hours, and the minimum and maximum duration of pain were 0 and 12 hours (pain duration did not follow normal distribution (*P* = 0.0001) (Table 1).

There were no reports of pain at 24 and 48 hours after the intervention. The frequency distribution of pain duration in the two groups was not statistically significant. In each group, the pain score decreased significantly over time. In the methylprednisolone group, unlike the dexamethasone group, the pain was significantly reduced 12 hours after the intervention compared with 6 hours after the intervention (*P* value <0.05).

## 4. Discussion

A flare-up is a postoperative pain and/or swelling of the facial soft tissues and the oral mucosa that starts after endodontic treatment of the involved teeth. It occurs within a few hours or a few days after the root canal therapy when clinical manifestations (tooth pain during function or by itself) are greatly expressed, and the patient requires urgent treatment sooner than the scheduled appointment. The etiology of the postendodontic flare-up involves mechanical, chemical, and microbial factors [20].

Despite modern root canal treatment methods and new rotary systems, flare-up remains a major problem for all dentists [21].

It occurs particularly for the patients exhibiting preoperative pain, and up to 80% of these patients may experience it. Flare-up rarely lasts longer than 72 h and usually is not so severe that it cannot be managed by NSAID agents [22].

Various categories of drugs have been prescribed for the management of this condition, including corticosteroids. Corticosteroids include dexamethasone and methylprednisolone, which reduce pain after endodontic treatment. Steroids bind to the glucocorticoid receptor, inhibiting proinflammatory signals and promoting anti-inflammatory signals [23]. Dexamethasone inhibits production through multiple cellular factors that are important for generating the inflammatory response [24].

In the present study, VAS score data showed that infiltration injection of dexamethasone and methylprednisolone had no statistically significant effect at 6 hours after the intervention in the two groups and this result is consistent with some studies [18, 25]. At 24 and 48 hours after treatment, there was no pain in either group. The effect of dexamethasone pretreatment on pain after endodontic treatment was consistent, so in 24 and 48 hours after treatment, more than 90% of patients did not have pain. Jalalzadeh et al. [17] in 2010 showed that 85% of patients receiving prednisolone had no pain. Also, Marshall and Walton [26] in 1984 indicated that more than 90% of patients who received steroid injection (dexamethasone) had analgesia or low pain within 24 and 48 hours after treatment. However, in Shahriari et al.'s study [27] on the effect of topical injection of dexamethasone on posttreatment pain after multistage RCT in 24 and 48 hours, 63.3% and 70% analgesia, respectively, occurred which is slightly consistent with the present study.

Sharma et al. in their study suggested that preoperative administration of SAID resulted in significantly less postoperative endodontic pain at all time intervals and preoperative oral administration of dexamethasone reduces pain postoperatively [28].

Another finding of this study is that, in each group, the pain score decreased considerably over time. Some studies are in agreement with this result [25, 29, 30]. The study by Mehrvarzfar et al. [18] in 2016 on the effect of intraligament injection of dexamethasone on pain after endodontic treatment is consistent with the present study in that patients' pain scores were significantly reduced over time. Praveen et al. [31]compared the effect of pretreatment with ketorolac and prednisolone on postoperative pain after endodontic treatment. In the prednisolone group, there was a significant reduction in pain over time.

In the present study, the mean pain score before the intervention and 6 hours after the intervention in each group was reduced. This result is consistent with some studies [24, 32].

A comparison of pain scores between 6 and 12 hours showed that the percent of patients that recorded no pain in the methylprednisolone group is more than that in the dexamethasone group. It could be because of its shorter half-life when compared with dexamethasone. Methylprednisolone requires less time to diffuse across cell membranes and alter gene transcription. Such an effect could not be observed at other time points. This could be explained by the fact that the intensity of postoperative endodontic pain decreased with time [32].

This study also has some limitations, such as sample size and lack of placebo group. Future randomized clinical trials with placebo group are required to obtain more accurate results.

## 5. Conclusions

There was no significant difference between infiltrated injections of dexamethasone and methylprednisolone in reducing pain after the endodontic treatment in necrotic teeth. Therefore, the use of any of these drugs to reduce pain after endodontic treatment is recommended.

## Figures and Tables

**Table 1:** Association of visual analogue score with group A and group B at 6, 12, 48, and 72 h.

Pain	Group A (*n* = 40) dexamethasone	Group B (*n* = 40) methylprednisolone	*P* value
*After 6 hours*			
No pain	34 (85%)	32 (80%)	0.51
Mild pain	4 (10%)	7 (17.5%)	
Moderate pain	1 (2.5%)	1 (2.5%)	
Severe pain	1 (2.5%)	0 (0%)	

*After 12 hours*			
No pain	37 (92.5%)	38 (95%)	1
Mild pain	3 (7.5%)	2 (5%)	
Moderate pain	0 (0%)	0 (0%)	
Severe pain	0 (0%)	0 (0%)	

*After 24 hours*			
No pain	40 (100%)	40 (100%)	∗
Mild pain	0 (0%)	0 (0%)	
Moderate pain	0 (0%)	0 (0%)	
Severe pain	0 (0%)	0 (0%)	

*After 48 hours*			
No pain	40 (100%)	40 (100%)	∗
Mild pain	0 (0%)	0 (0%)	
Moderate pain	0 (0%)	0 (0%)	
Severe pain	0 (0%)	0 (0%)	

^∗^No statistics are computed because pain in 24 and 48 hours is a constant.

## Data Availability

The datasets used and/or analyzed during the current study are available from the corresponding author upon request.
